# Optimization of the image acquisition procedure in low-field MRI for non-destructive analysis of loin using predictive models

**DOI:** 10.7717/peerj-cs.583

**Published:** 2021-06-07

**Authors:** Daniel Caballero, Trinidad Pérez-Palacios, Andrés Caro, Mar Ávila, Teresa Antequera

**Affiliations:** 1 Department of Computer Systems and Telematics Engineering, University of Extremadura, Caceres, Spain; 2 Faculty of Sciences and University of Copenhagen, Copenhagen, Denmark; 3 Food Technology Department and University of Extremadura, Caceres, Spain

**Keywords:** Data Mining, Feature extraction, MRI, Predictive models, Optimization, Central Composite Design

## Abstract

The use of low-field magnetic resonance imaging (LF-MRI) scanners has increased in recent years. The low economic cost in comparison to high-field (HF-MRI) scanners and the ease of maintenance make this type of scanner the best choice for nonmedical purposes. However, LF-MRI scanners produce low-quality images, which encourages the identification of optimization procedures to generate the best possible images. In this paper, optimization of the image acquisition procedure for an LF-MRI scanner is presented, and predictive models are developed. The MRI acquisition procedure was optimized to determine the physicochemical characteristics of pork loin in a nondestructive way using MRI, feature extraction algorithms and data processing methods. The most critical parameters (relaxation times, repetition time, and echo time) of the LF-MRI scanner were optimized, presenting a procedure that could be easily reproduced in other environments or for other purposes. In addition, two feature extraction algorithms (gray level co-occurrence matrix (GLCM) and one point fractal texture algorithm (OPFTA)) were evaluated. The optimization procedure was validated by using several evaluation metrics, achieving reliable and accurate results (*r* > 0.85; weighted absolute percentage error (WAPE) lower than 0.1%; root mean square error of prediction (RMSEP) lower than 0.1%; true standard deviation (TSTD) lower than 2; and mean absolute error (MAE) lower than 2). These results support the high degree of feasibility and accuracy of the optimized procedure of LF-MRI acquisition. No other papers present a procedure to optimize the image acquisition process in LF-MRI. Eventually, the optimization procedure could be applied to other LF-MRI systems.

## Introduction

Many scientific studies are based on computer vision algorithms and magnetic resonance imaging (MRI), mainly focusing on medical (and veterinary) radiology for diagnostic imaging. For many years, these techniques have also been utilized for food inspection and monitoring to guarantee food safety and product quality (Bajd et al., 2016b; Bajd et al., 2016a; Bertram et al., 2005; Fantazzini et al., 2009; García-García et al., 2019; Hansen et al., 2008; Pérez-Palacios et al., 2010; Pérez-Palacios et al., 2011; Pérez-Palacios et al., 2014; Ishiwatari, Fukuoka & Sakai, 2013; Lee et al., 2015; Mahmoud-Ghoneim et al., 2005; Portanguen et al., 2014; Shaarani, Nott & Hall, 2006). Multidisciplinary teams have been involved in these scientific studies, from computer vision, MRI, data analysis, and different areas of knowledge: medicine, veterinary medicine, and food technology. However, the general procedure for optimizing the MRI acquisition procedure and computer vision algorithms for a specific purpose has not been revised. This paper presents a generic procedure to optimize the analysis of a meat product (specifically pork loin) by MRI and computer vision.

In the case study presented, computer vision techniques and MRI were proposed in recent years as an alternative methodology to traditional methods of analysis of meat and meat derivatives (mainly hams and loins) since they are nondestructive, noninvasive, nonintrusive, nonionizing, and innocuous (Ávila et al., 2018; Ávila et al., 2019; Caballero et al., 2017d; Caballero et al., 2018b). Feature extraction algorithms were employed to obtain numerical characteristics from MRI (Ávila et al., 2019; Caballero et al., 2017d; Caballero et al., 2018b), forming datasets of attributes extracted from the images.

Data mining methods were also included in the usual procedure to process data extracted from MRI, to classify products, to detect anomalies, to study quality parameters, or even to predict the quality of the products (Ávila et al., 2018; Ávila et al., 2019; Caballero et al., 2017d; Caballero et al., 2018b; Pérez-Palacios et al., 2017). In general, the general procedure for the analysis of meat products by MRI consists of three main steps: image acquisition, feature extraction, and data analysis.

For image acquisition, high-field magnetic resonance imaging (HF-MRI) scanners have been frequently utilized in meat and meat product research (Bajd et al., 2016b; Bajd et al., 2016a; Bertram et al., 2005; Fantazzini et al., 2009; García-García et al., 2019; Hansen et al., 2008; Pérez-Palacios et al., 2010; Pérez-Palacios et al., 2011; Pérez-Palacios et al., 2014; Ishiwatari, Fukuoka & Sakai, 2013; Lee et al., 2015; Mahmoud-Ghoneim et al., 2005; Portanguen et al., 2014; Shaarani, Nott & Hall, 2006). However, HF-MRI scanners are expensive and require high maintenance costs, such as liquid helium for refrigeration (Ladd et al., 2018; Feig, 2011). This kind of device is especially suitable for medical (and veterinary) purposes. In contrast, the use of low-field magnetic resonance imaging (LF-MRI) scanners has increased in food technology applications for the evaluation of quality parameters of meat products (Bernau et al., 2015; Manzoco et al., 2013; Monziols et al., 2006; Torres et al., 2019) in recent years since they are less expensive than HF-MRI scanners and do not have high maintenance costs (they can be refrigerated simply by air). The LF-MRI magnetic field varies from 0.15 to 0.50 T (Ladd et al., 2018), and consequently, these scanners produce lower-quality images (low signal-to-noise ratio) (Ladd et al., 2018). This disadvantage poses a challenge to obtaining images that can be computationally analyzed and requires the optimization of procedures for image acquisition (Pérez-Palacios et al., 2017).

From the acquisition sequence point of view, three methods were tested in LF-MRI studies of meat and meat products (spin echo (SE), gradient echo (GE) and turbo 3D (T3D)) (Caballero et al., 2017d; Caballero et al., 2018b; Pérez-Palacios et al., 2017). In general, SE led to sharper and better-defined images and achieved higher correlation coefficients than GE and T3D for the prediction of quality parameters of meat products (Caballero et al., 2018b; Pérez-Palacios et al., 2017).

In relation to relaxation times, MRI can be weighted on T1 and T2 relaxation times. T1 or spin-lattice relaxation time is the time from longitudinal magnetization until the equilibrium value of the relaxation times has been exponentially decreased. T2 or spin-spin relaxation time describes the same process for transverse magnetization. There are also other critical parameters in addition to the MRI contrast, such as echo time (TE) and repetition time (TR), that must be established for image acquisition (Pérez-Palacios et al., 2017). TE is the time from the center of the radio frequency pulse to the center of the echo and principally controls the amount of T2. TR represents the length of time between corresponding consecutive series of pulses and echoes and determines the longitudinal magnetization recovered between each pulse (Hendrick, 2005; Stark & Bradley, 1999).

Regarding feature extraction, algorithms based on textures (gray level co-occurrence matrix (GLCM), gray level run length matrix (GLRLM) and neighboring gray level dependence matrix (NGLDM)) and fractals (classical fractal algorithm (CFA), fractal texture algorithm (FTA) and one point fractal texture algorithm (OPFTA)) have been principally applied (Caballero et al., 2018b; Caballero et al., 2017c; Galloway, 1975; Haralick, Shanmugam & Dinstein, 1973; Mandelbrot, 1982; Siew, Hodgson & Wood, 1988). Thus, texture algorithms measure gray levels and integrate matrices based on second-order statistics (Sonka, Hlavac & Boyle, 1999), while fractals allow the identification of recurring patterns (Mandelbrot, 1982). In previous studies (Caballero et al., 2017d; Caballero et al., 2018b; Pérez-Palacios et al., 2017; Caballero et al., 2017a; Caballero et al., 2018a), GLCM and OPFTA were identified as the best options.

For the data analysis, common statistical tools, such as Pearson’s correlation coefficients, analysis of variance (ANOVA) or principal component analysis (PCA) have been applied, showing promising results (Pérez-Palacios et al., 2010; Bro & Smilde, 2014; Cernadas et al., 2005). In recent years, data mining techniques have also been employed. Data mining is a nontrivial process of obtaining knowledge and potentially useful information from data stored in repositories (Fayyad, Piatetsky-Shapiro & Smyth, 1996). Data mining technologies present important threats to the security and privacy of data (Xu et al., 2014). Concerns are raised when analytics deploy artificial intelligence (AI) techniques, including machine-learning algorithms (Rastogi, Gloria & Hendler, 2015). The preservation of privacy in data mining is also discussed in Aldeen, Salleh & Razzaque (2015). In addition, privacy-preserving data mining mechanisms are reviewed in Sangeetha & Sudha Sadasivam (2019). In this paper, data mining algorithms utilized in the developed system preserve the security and privacy necessary to guarantee the validity of the results.

Considering data analysis, many papers are based on machine learning and deep learning algorithms. For example, a special neural network for cybersecurity purposes is employed in Sarkar et al. (2021), while machine learning algorithms are applied in Muhammad et al. (2021), where the correlation coefficient r is applied as the evaluation metric, quite similar to the metric presented in this paper. In the same way, convolutional neural networks (CNNs) have also been employed as deep learning algorithms for multiple purposes, for example, to detect anomalies in automated vehicles (Javed et al., 2021) or classification tasks (Gadekallu et al., 2021; Vasan et al., 2020).

There are many published clinical applications of the use of data mining in MRI studies (Itoni, Lecron & Fortemps, 2019).

Since the MRI-extracted features depend on the image quality, the optimization of the image acquisition parameters is a key aspect for accurately determining the quality parameters of the meat products. In this case, different data mining techniques (such as multiple linear regression (MLR), partial least squares (PLS) or isotopic regression (IR)) have been tested in MRI studies to predict the quality characteristics (Ávila et al., 2019; Caballero et al., 2017d; Caballero et al., 2018b; Pérez-Palacios et al., 2017; Caballero et al., 2017a; Caballero et al., 2018a).

In Ávila et al. (2019), many data mining techniques were tested, obtaining different performances as a function of the quality parameter of loins. Moreover, MLR was applied to evaluate the quality characteristics of beef (Song, Kim & Lee, 2002) and lamb (Cortez et al., 2006). As a result, MLR was selected as a data mining technique to optimize the procedure of the analysis of meat products by using LF-MRI.

Optimizing the MRI acquisition procedure in MRI is not a trivial task, mainly because it depends on multiple parameters and purposes and on the type of MRI device (HF-MRI vs. LF-MRI).

HF-MRI scanners have been frequently employed in meat and meat products based on ham and loins (Bajd et al., 2016b; Bajd et al., 2016a; Bertram et al., 2005; Fantazzini et al., 2009; García-García et al., 2019; Hansen et al., 2008; Pérez-Palacios et al., 2010; Pérez-Palacios et al., 2011; Pérez-Palacios et al., 2014), beef (Ishiwatari, Fukuoka & Sakai, 2013; Lee et al., 2015; Mahmoud-Ghoneim et al., 2005; Portanguen et al., 2014) or chicken (Shaarani, Nott & Hall, 2006). However, there are very few studies that present the use of LF-MRI applied to food technology. In previous works, LF-MRI scanners were used to evaluate the quality parameters of hams and loins (Bernau et al., 2015; Manzoco et al., 2013; Monziols et al., 2006; Torres et al., 2019). Likewise, few studies have shown any type of optimization in the image acquisition process of an LF-MRI scanner. For LF-MRI devices, the acquisition sequence was revised in Pérez-Palacios et al., (2017), whereas the performance of some feature algorithms was presented in Caballero et al. (2018b); Pérez-Palacios et al. (2017).

In addition, the type of scanner was compared in Caballero et al. (2021), Antequera et al. (2021), where the advantages and disadvantages of the HF-MRI and LF-MRI scanners were presented. In St-Jean et al. (2020), Balsigier et al. (2020), the signal-to-noise ratio was optimized to create a stable and reproducible reconstruction algorithm.

Other authors focus on different parameters to optimize the MRI acquisition procedure. In this way, the field of view (FOV) was optimized in Aoyama et al. (2018), Walker et al. (2014) to reduce the geometric distortion for the configuration of the MRI. Similarly, the position of the sample was reviewed in Bajd et al. (2016b), Bernau et al. (2015), achieving the best position as a function of the coil used.

In this paper, the image acquisition procedure for an LF-MRI scanner was optimized. The efforts in this paper focused on optimizing the three fundamental parameters in the image acquisition process, such as relaxation times (T1 and T2), TR, and TE, in a LF-MRI device. To achieve this optimization, ANOVA was conducted to optimize the relaxation times T1 and T2. Similarly, the response surface methodology (RSM) was used to adjust the TE and TR because this method considers the relationships between more than one measure and the remaining measures of the method (Leardi, 2009). In addition, two feature extraction algorithms (GLCM and OPFTA) were tested in this paper to select the best option for the feature extraction phase.

**Table 1:** Summary of the optimized parameters. Comparison of different methods.

Procedure step	Optimized parameter	MRI scanner	Ref.	Year
Image acquisition	Field-of-view (FOV)	HF	Molano et al. (2012)	2014
Data analysis	Data mining methods (MLR and IR)	HF	Bajd et al. (2016a)	2014
Image acquisition	Sample position	HF	Caballero et al. (2017d)	2015
Image acquisition	Sample position	HF	Aggarwal & Agrawal (2012)	2016
Image acquisition & Feature extraction	Acquisition sequence (SE, GE and T3D) Feature extraction methods (GLCM, GLRLM and NGLDM)	LF	Caballero et al. (2017c)	2017
Image acquisition	Field-of-view (FOV)	HF	Menéndez et al. (2018)	2018
Feature extraction	Feature extraction methods (CFA, FTA, GLCM, GLRLM, LBP, NGLDM and OPFTA)	LF	Caballero et al. (2017b)	2018
Data analysis	Data mining methods (LM, Penalized, KrlsRadial, Foba, avNNet, GRNN, Kelm, Dlkeras, SVR, M5, Cubist, Earth, BagEarth, GBM, GAMBoost, RF, Boruta, RRF, CForest, Extratrees, QRF, Rqlasso, BRNN, Bartmachine, GaussPrPoly, LARS, PPR, ENET)	HF/LF	Caballero et al. (2017a)	2019
Image acquisition	Signal-to-noise ratio (S/N)	HF	Mandelbrot (1982)	2020
Image acquisition	Signal-to-noise ratio (S/N)	HF	Manzoco et al. (2013)	2020
Image acquisition	Type of scanner (HF and LF)	HF/LF	Lufkin (1998)	2021
Image acquisition	Type of scanner (HF and LF)	HF/LF	Mahmoud-Ghoneim et al. (2005)	2021
Image acquisition & Feature extraction	Relaxation times (T1 and T2) Echo time (TE) Repetition time (TR) Feature extraction methods (GLCM and OPFTA)	LF	This paper	2021

Table 1 shows a summary of the parameters optimized in the MRI procedure, considering the loin samples in all the experiments. To the best of our knowledge, no studies have been performed to optimize the image acquisition procedure of an LF-MRI scanner.

The main objective of this study is the optimization of the most critical tunable parameters (relaxation times (T1 and T2), TR, and TE) of the LF-MRI scanner to provide a valid and usable optimization procedure for multiple purposes. In particular, in this study, the optimization procedure is oriented to the prediction of the physicochemical characteristics of fresh and dry-cured loins.

The main contributions of this paper can be summarized as follows: (i) a procedure to optimize the image acquisition process in LF-MRI is presented; (ii) the optimization procedure is adapted for a practical application (nondestructive analysis of loins); (iii) the performance of feature extraction algorithms has been tested; (iv) no other papers present a procedure to optimize the image acquisition process in LF-MRI; and (v) the optimization procedure presented in this paper can be easily replicated in other environments or for other purposes.

The paper has been organized as follows: ‘Materials and Methods’ exposes the materials and methods that have been carried out, including the experimental design of the study; ‘Results and Discussion’ presents and discusses the results of the two experiments, showing the achievements obtained by the optimization processes; and ‘Conclusions and Future Works’ exposes the conclusions and future trends.

## Material and Methods

**Figure 1:**
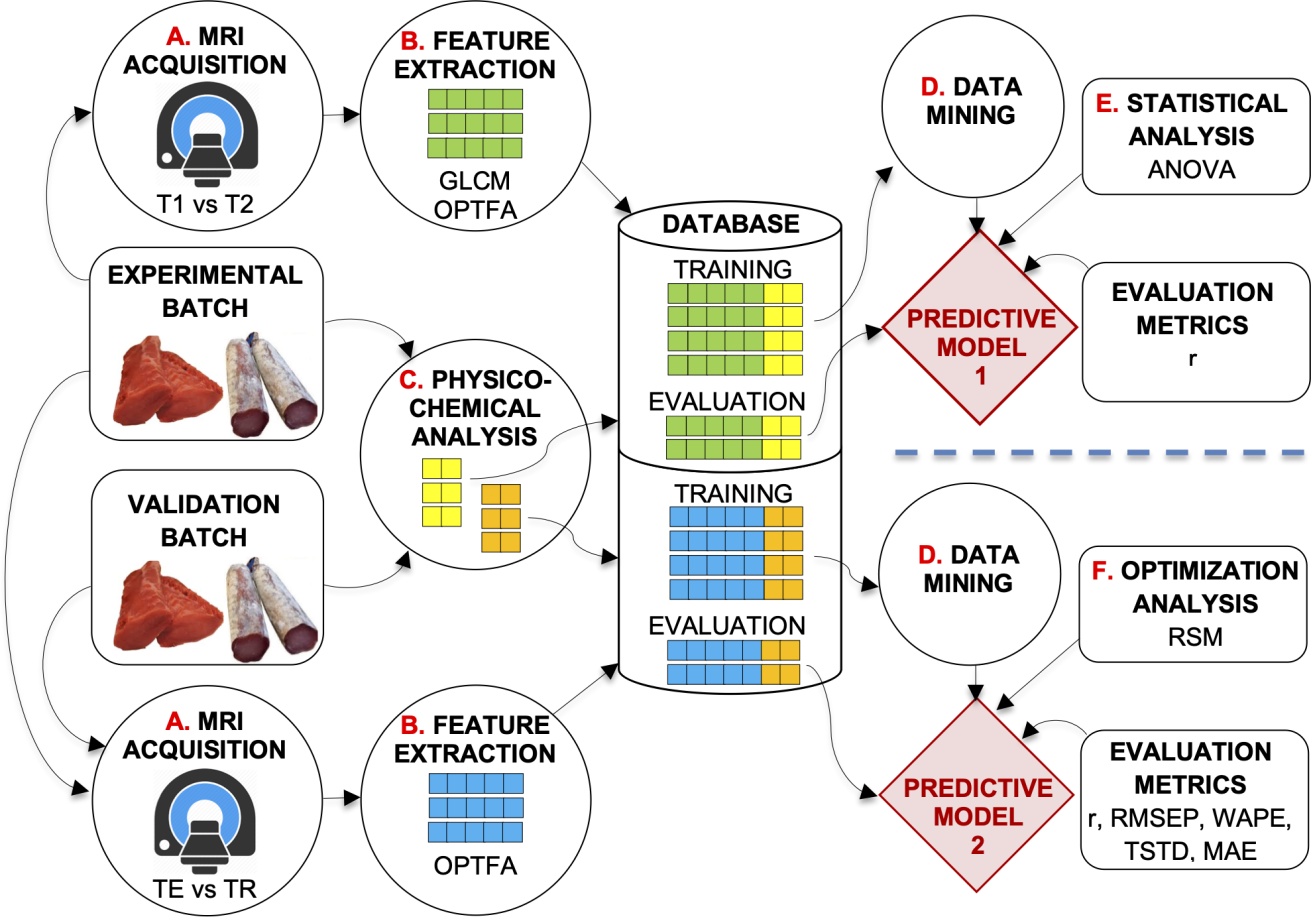
Description of the system developed. Experimental design of the study.

This study was carried out on a dataset of 12,528 MRIs of pork loins (half of them as fresh products and the other half as dry-cured products). The fresh loins were frozen (−18 ±  2 °C) and thawed the day before they were analyzed. The dry-cured loins were stored at ambient temperature (20 ± 2 °C). Figure 1 shows the experimental design.

Two experiments were carried out with the optimization batch (Fig. 1). In the first experiment (at the top of Fig. 1), SE-T1-weighted vs. SE-T2-weighted contrasts and GLCM vs. OPFTA algorithms were compared, selecting the options that produce the best predictions. Thus, the optimal relaxation time parameter and optimal feature extraction algorithm could be determined.

The second experiment aimed to optimize TE and TR acquisition parameters by applying the RSM (at the bottom of Fig. 1).

The general procedure for image acquisition, feature extraction, and physicochemical analysis was similar in these experiments. First, loins were MRI scanned (Fig. 1, process A). Second, images were analyzed (Fig. 1, process B), extracting features by means of the best computer vision algorithms according to  Caballero et al. (2017d), Caballero et al. (2018b), Pérez-Palacios et al. (2017), Caballero et al. (2017a), Caballero et al., (2018a). After the image acquisition procedure, the loins were physicochemically analyzed (Fig. 1, process C), determining quality parameters such as water activity, pH, moisture, lipid and salt content, and instrumental color L^∗^. All the data obtained were stored in a dataset repository (Database, in Fig. 1). Finally, predictive techniques of data mining were applied to the datasets (Fig. 1, process D) to obtain predictive models. Other analyses affected the obtained models (Fig. 1, processes E and F), which were tested by means of several validation metrics.

As a result of the first experiment, predictive model 1 allowed the optimization of SE sequence acquisition (T1-weighted vs. T2-weighted), as well as identification of the best feature extraction algorithm (GLCM vs. OPFTA). T1 and OPFTA resulted in the best combination, according to the first experiment and the results of the “Results and Discussion” section of this paper. This combination was employed in the second experiment to optimize the TE and TR acquisition parameters.

In this way, the relaxation times (T1 and T2), TR, and TE of the LF-MRI scanner were optimized for the image acquisition process. To mention some gaps, other parameters could also be optimized, such as FOV, slice thickness, or number of repetitions. However, for the practical application proposed in this work, it was enough to optimize the parameters considered. Regardless, the optimization of any other parameter could be solved by following the same optimization model proposed in this paper.

To perform the optimization procedure, a computational system was implemented according to the experimental design. The system complied with the secure software development guidelines specified in (Sancho, Caro & Rodríguez (2020) and the considerations on security risk estimations presented in Sancho et al. (2020). They contributed to the development of a secure system, especially considering the importance and significance of research related to food technology in relation to food safety.

### MRI acquisition

MRI was performed at the University of Extremadura (Cáceres, Spain). A LF-MRI scanner (ESAOTE VET-MR E-SCAN XQ 0.18 T) with a hand/wrist coil was utilized. SE weighted on T1 and T2 were selected for sequence acquisition. In the first experiment, standard acquisition sequence parameters established by the MRI scanner manufacturer were employed (Pérez-Palacios et al., 2008; ESAOTE SpA., 2007). In the case of the SE-T1-weighted sequence, the standard acquisition parameters (ESAOTE SpA., 2007) were as follows: TE: 26 ms; TR: 630 ms; FOV: 150 × 150 mm^2^; slice thickness: four mm; flip angle: 90°; matrix size: 256 ×204; phase encode: 204; number of repetitions: five per sample; 29 slices per loin were obtained; and MRI acquisition took 50 min for each loin.

For the SE-T2-weighted sequence, the standard acquisition parameters established by the MRI scanner manufacturer (Pérez-Palacios et al., 2008; ESAOTE SpA., 2007) were as follows: TE: 80 ms; TR: 1,800 ms; FOV: 150 × 150 mm^2^; slice thickness: four mm; flip angle: 90°; matrix size: 256 ×204; phase encode: 204; number of repetitions: three per sample; 29 slices per loin were obtained and the MRI acquisition took 75 min for each loin.

In the second experiment, TE and TR parameters were optimized in the SE-T1-weighted acquisition sequence, because this was the best acquisition sequence determined in the first experiment. The remaining parameters for image acquisition were established according to standard parameters given by the MRI scanner manufacturer (Pérez-Palacios et al., 2008; ESAOTE SpA., 2007).

### Feature extraction

On the MRI dataset of loins, the best feature extraction algorithms were applied to extract feature vectors from the images (Caballero et al., 2017d; Caballero et al., 2018b; Pérez-Palacios et al., 2017; Caballero et al., 2017a; Caballero et al., 2018a). In Caballero et al. (2018b), different feature extraction algorithms were compared, presenting the lower computational cost of GLCM and OPFTA (O (n^2^)). The OPFTA achieved the best correlation coefficients (*r* > 0.75) and lower computation times (<50 ms). Therefore, these two algorithms were selected as feature extraction algorithms for the experiments presented in this paper: GLCM and OPFTA were utilized in experiment 1, whereas OPFTA was selected for the second experiment.

Both algorithms required, as a previous step, selection of the largest area rectangles inscribed on the image closed contour (Caro et al., 2004; Molano et al., 2012), which are referred to as regions of interest (ROIs). These ROIs must be rectangular for applying the algorithms (Pan, Li & Wei, 2007). There were no additional previous requirements for the application of the image analysis algorithms.

GLCM was performed by counting the number of times that each pair of gray levels (*i*, *j*) occurred at a given distance *“d”* in all directions. In this matrix, each item *p(i, j)* indicates the number of times that two neighboring pixels separated by distance *d* (*d* = 1 in this case) occur on the image—the first pixel with gray level *i* and the second pixel with gray level *j*—in all 2D directions: 0°, 45°, 90° and 135°. These co-occurrences are accumulated into a single matrix, from which ten statistical features are extracted: energy (ENE), entropy (ENT), correlation (COR), Haralick’s correlation (HC), inverse difference moment (IDM), inertia (INE), cluster shade (CS), cluster prominence (CP), contrast (CON), and dissimilarity (DIS) (Haralick, Shanmugam & Dinstein, 1973).

After ROI selection (Molano et al., 2012), each rectangle was divided into smaller rectangles of 32 ×32 pixels: which were called mini-ROI. At this point, the OPFTA fractal values were obtained from these mini-ROIs by selecting the value for the box size equal to 8 (Caballero et al., 2017b). Next, these values were gathered to create a matrix, in which each cell of the matrix represents one mini-ROI from the image. Seven features were calculated on the matrix by applying second-order statistics: uniformity (UNI), ENT, COR, homogeneity (HOM), INE, CON and efficiency (EFI) (Aggarwal & Agrawal, 2012; Peckinpaugh, 1991).

### Physicochemical dataset acquisition

The physicochemical analyses carried out on the loins consisted of determining the following parameters:

• Water activity was determined by using the Lab Master-aw system (NOVASINA AG, Lachen, Switzerland) after calibration.
• pH was determined with a glass electrode pH meter model CyberScan pH 510 (Eutech instruments, Illkirch, France) that tests a 10 ml volume. The pH meter was calibrated with commercial buffer solutions (Crison, Barcelona, Spain) at pH 4.0, 7.0 and 9.0 prior to use.
• Moisture content was determined by the official method (Association of Official Analytical Chemist, 2000) (Ref. 935.29) at 100 ±  2 °C.
• The lipid content was determined gravimetrically with chloroform:methanol (2:1, v/v) according to the method described in Pérez-Palacios et al. (2008).
• Instrumental color was determined by using a Minolta CR-300 (Minolta Camera Corp., Meter Division, Ramsey, New Jersey, U.S.A.) with illuminant D65 at a 0° standard observer and a 2.5 cm^2^ port/viewing area. The following color coordinates were determined: lightness (L ^∗^), redness to green (a ^∗^) and yellowness to blueness (b ^∗^). The colorimeter was standardized before use with a white tile that has the following values: L ^∗^ = 93.5, a ^∗^ = 1.0 and b ^∗^ = 0.8.
• The salt content was determined volumetrically in dry-cured loins by using the official method (Association of Official Analytical Chemist, 2000) (Ref. 971.19).

All determinations were performed in triplicate.

### Data mining predictive models

Predictive techniques of data mining were utilized to create predictive models from current data using trend analysis (Wu et al., 2008). In this study, MLR was applied to model the linear relationship between a target variable and more independent prediction variables (Rencher & Christiansen, 2012). The M5 method of attribute selection was applied, stepping through the attributes, with the smallest standardized coefficient removed until no improvement was observed in the estimation of the error. A ridge value of 1 × 10^−4^ was also applied, and the remaining parameters were configured with the following values: batch size = 100; Debug = False; Do Not Check Capabilities = False; Eliminate Colinear Attributes = False; Minimal = False; Num Decimal Places = 4; Output Additional Stats = False; and Use QR Decomposition = False).

The computer system has been developed in the programming languages C/C++ and Java. The machine learning models are based on the algorithms developed in R, Python and Weka, using their APIs.

The estimation procedure was performed by a 10-fold cross validation method (Dietterich, 1998), where the dataset was divided into ten partitions of equal size. One subset was tested each time, and the remaining data were used to fit the model. This process was repeated until all subsets were tested. Although this method requires 10 repetition analyses, it is a robust method (R. Grossman et al., 2010).

Different performance metrics have been used to compare the predicted values and actual values. In our experiments, the real values were obtained by physicochemical analysis (process C in Fig. 1, explained in the previous section C), and the predicted values were obtained using predictive models 1 and 2 (using the data mining algorithms discussed in this section).

Metrics such as the correlation coefficient, root mean square error of prediction (RMSEP), mean absolute error (MAE), true standard deviation (TSTD), and weighted absolute percentage error (WAPE) were applied to evaluate the performance of the model. Table 2 briefly describes the metrics used in this research and their mathematical representation.

**Table 2:** Metrics used to validate the predictive models. Evaluation metrics.

Equation	Formula	Values
(1)	$r=\sqrt{ \frac{{\mathop{\sum }\nolimits }_{i=1}^{n}({f}_{i}-y)^{2}}{{\mathop{\sum }\nolimits }_{i=1}^{n}({y}_{i}-y)^{2}} }$	*f*_*i*_: predicted value *y*_*i*_: real value *y*: average value *n*: number of samples
(2)	$RMSEP \left( \text{%} \right) =\sqrt{ \frac{1}{n} {\mathop{\sum }\nolimits }_{i=1}^{n}({f}_{i}-{y}_{i})^{2}}x100$	*f*_*i*_: predicted value *y*_*i*_: real value *n*: number of samples
(3)	$MAE= \frac{1}{n} {\mathop{\sum }\nolimits }_{i=1}^{n} \left\vert {f}_{i}-{y}_{i} \right\vert $	*f*_*i*_: predicted value *y*_*i*_: real value *n*: number of samples
(4)	$TSTD= \frac{1}{N} {\mathop{\sum }\nolimits }_{i=1}^{N}\sqrt{ \frac{1}{{M}_{k}-1} {\mathop{\sum }\nolimits }_{j=1}^{{M}_{j}}({d}_{ij}-{d}_{i})^{2}}$	*N*: number of samples *M*_*j*_: number of true measurements *d*_*ij*_: *j*^*th*^ true measurement of the sample *id*_*i*_: mean value of all measurements for sample *i*
(5)	$WAPE \left( \text{%} \right) = \frac{100\cdot {\mathop{\sum }\nolimits }_{i=1}^{n} \left\vert {f}_{i}-{y}_{i} \right\vert }{{\mathop{\sum }\nolimits }_{i=1}^{n}{f}_{i}} $	*f*_*i*_: predicted value *y*_*i*_: real value *n*: number of samples

The correlation coefficient r (Table 2, equation 1), which is one of the most common metrics in classification and regression, was used to evaluate the goodness of fit of the prediction of the quality parameters and for its validation, according to the rules given by Colton (Colton, 1974).

Moreover, the RMSEP (Table 2, equation 2) was also used to evaluate the prediction results (Ávila et al., 2019; Hyndman & Koehler, 2006). The RMSEP measures the error between the real values and the predicted values. This measure is commonly used to assess the predictive ability of the models since it is a constant measure for prediction. Real and predicted values were also compared by MAE (Table 2, equation 3). TSTD (Table 2, equation 4) was applied to evaluate the mean dispersion of the true measurements, and WAPE (Table 2, equation 5) was used to measure the mean dispersion of the computer prediction values around the attribute (Ávila et al., 2019).

In all the experiments, the correlation coefficient f (Table 2, equation 1) was applied to validate the predictive models, following Colton’s rules, where correlations greater than 0.75 indicate good to excellent results.

### Statistical analysis

The correlation coefficient between extracted features from images acquired by T1-weighted and T2-weighted MRI contrasts and physicochemical characteristics of loins was calculated. The effect of the MRI contrast on extracted features from the GLCM and OPFTA and differences between the results from the MRI analysis and the results from the physicochemical analysis were analyzed by a one-way ANOVA to compare the mean values.

### Optimization analysis

The RSM was employed to optimize the TE and TR parameters of the LF-MRI acquisition of fresh and dry-cured loins. A full factorial central composite design (CCD) for each type of loin was applied. The design consists of a complete 2^3^ factorial design with five center points and one axial point of the axis of each design variable at a distance of *α* = 1 from the design center. Table 3 shows coded (as a function of *α*) and uncoded (real values) TE and TR (independent variables), which were adjusted between 18 ms and 26 ms and between 630 ms and 910 ms, respectively, considering the standard acquisition parameters established by the MRI scanner manufacturer (ESAOTE SpA., 2007). The complete design had 13 combinations (runs) of TE and TR, including 5 replicates of the center point (TE =22 ms, TR =770 ms). At each TE-TR combination, the response evaluated was the correlation coefficient (r) for the prediction equations of the physicochemical parameters (water activity, pH, moisture and lipid content, and instrumental color (L*), and in the case of the dry-cured loins, also the salt content).

**Table 3:** Coded and uncoded of the independent variables and responses obtained for the central composite design to optimize the spin echo acquisition parameters (Echo Time (TE), Repetition Time (TR)).

	Independent variables				Responses				
RUN	Coded		Uncoded		r				
	TE (ms)	TR (ms)	TE (ms)	TR (ms)	aW	pH	Moisture	Lipids	Color L^∗^
1	1	1	26	910	0.958	0.864	0.881	0.953	0.822
2	0	0	22	770	0.988	0.916	0.972	0.987	0.950
3	0	0	22	770	0.977	0.943	0.970	0.977	0.960
4	0	0	22	770	0.982	0.968	0.969	0.980	0.957
5	0	−1	22	630	0.980	0.962	0.965	0.979	0.958
6	0	1	22	910	0.983	0.967	0.969	0.982	0.962
7	−1	0	18	770	0.982	0.888	0.936	0.979	0.900
8	1	−1	26	630	0.976	0.970	0.966	0.975	0.962
9	−1	1	18	910	0.976	0.965	0.952	0.975	0.945
10	0	0	22	770	0.975	0.958	0.961	0.974	0.954
11	1	0	26	770	0.965	0.945	0.931	0.962	0.920
12	0	0	22	770	0.970	0.963	0.960	0.968	0.956
13	−1	−1	18	630	0.974	0.911	0.942	0.971	0.922

This finding is one of the highlights of the paper, because no other papers apply the RSM to optimize the sequence acquisition of LF-MRI scanners.

## Results and Discussion

### Optimization of the MRI acquisition parameters

**Figure 2:**
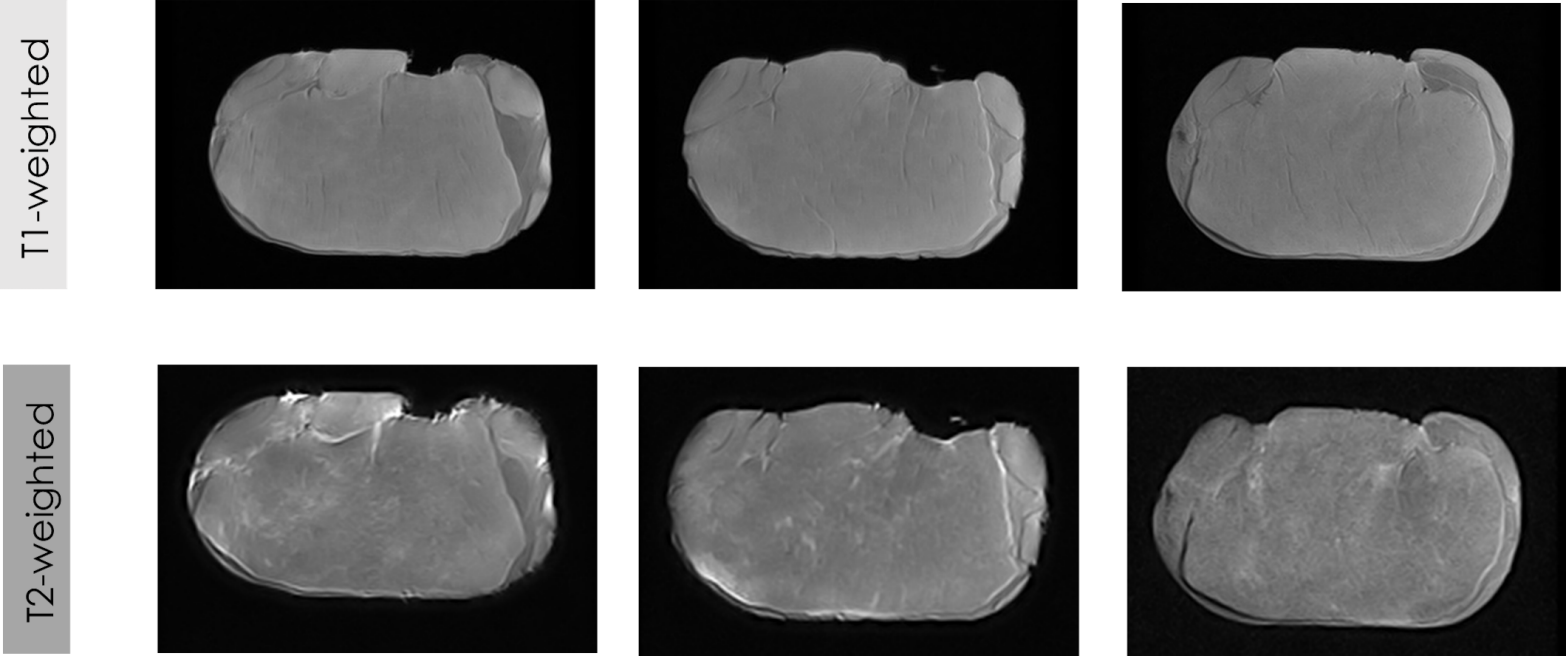
Some examples of LF-MRI images of pork loins. MRI of fresh loins acquired by LF-MRI applying SE weighted on T1 and T2.

The first trial of this study evaluated the influence of MRI contrast (T1-weighted vs. T2-weighted) and SE as an acquisition sequence for MRI acquisition. Figure 2 shows MRI images of fresh loins acquired by applying SE weighted on T1 and T2. In addition, this first experiment also tested the performance of the feature extraction algorithms (GLCM vs. OPFTA).

Visual differences in MRI can be reached depending on the contrast applied for the acquisition of the images (T1-weighted and T2-weighted). The gray color representing the muscle is slightly darker in the T1-weighted images than in T2-weighted images, while the white color of the fat is darker in the T1-weighted images than in the T2-weighted images. Thus, the different magnetizations produced by the different acquisition sequences (T1-weighted and T2-weighted) provide different values for the contrast of the MRI. The different magnetization of each contrast (Edelstein et al., 1983; Elster, 1988; Young, Burl & Bydder, 1986), which provides different levels of saturation and gray levels in the images, produces significant differences in all features from texture and fractals algorithms between T1-weighted images and T2-weighted images (*p* < 0.001). Previous studies also showed the influence of other parameters of the MRI acquisition, specifically the type of acquisition sequence (SE vs. GE vs. T3D), on the values of the extracted features (Caballero et al., 2017d; Pérez-Palacios et al., 2017; Caballero et al., 2017a).

**Figure 3:**
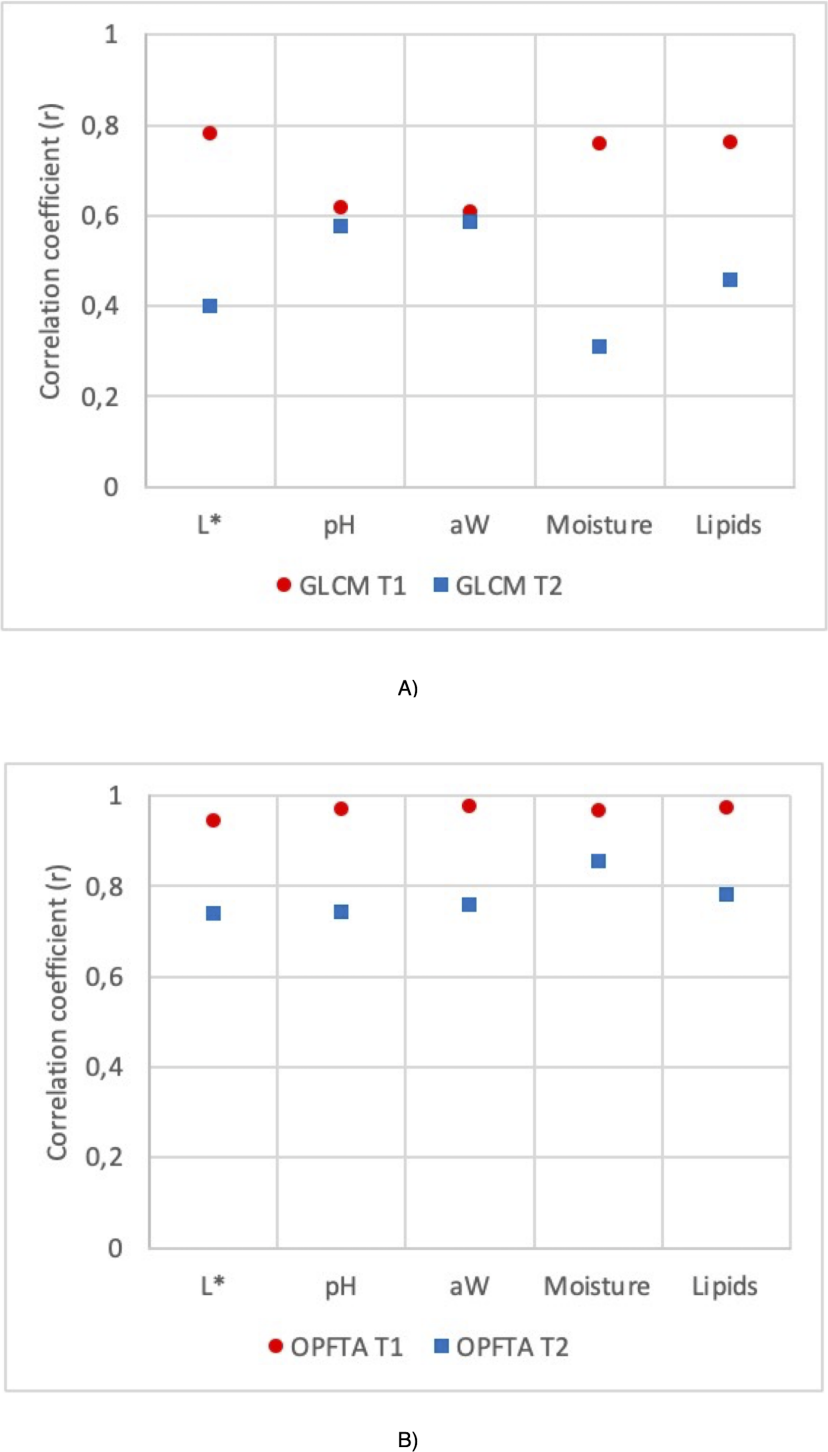
Correlation coefficient (r) for the GLCM and OPFTA feature texture algorithms. Correlation coefficients of physico-chemical parameters of fresh loins predicted by MRI applying T1-weighted and T2-weighted contrast, for the image acquisition, and GLCM (A) and OPFTA (B), as feature extraction algorithms.

Figure 3 shows the correlation coefficients of the physicochemical parameters of fresh loins predicted by MRI applying T1-weighted and T2-weighted contrast by using GLCM (Fig. 3A) or OPFTA (Fig. 3B). Higher correlation coefficients were reached with SE-T1-weighted contrast than with SE-T2-weighted contrast for all physicochemical parameters when applying both GLCM and OPFTA. In all cases, no significant differences were found (*p* > 0.05) between real values (physicochemical analysis) and predicted values from the different MRI analyses (SE-T1-weighted GLCM, SE-T2-weighted GLCM, SE-T1-weighted OPFTA and SE-T2-weighted OPFTA). Previous studies on meat applying T1-weighted and T2-weighted MRI contrasts (García-García et al., 2019; Manzoco et al., 2013; Fantazzini et al., 2005) pointed out that the application of both T1-weighted and T2-weighted contrasts was suitable for the analysis of meat, achieving better T1-weighted results than T2-weighted results in all cases. This major accuracy of T1-weighted imaging could be produced by the higher contrast and gray levels obtained with this MRI contrast (Elster, 1988). In the present study, MRI acquisition took less time when using SE-T1-weighted imaging (50 min) than SE-T2-weighted imaging (75 min), which also suggests the pertinence of SE-T1-weighted imaging, in concordance with other studies (Caballero et al., 2017d; Caballero et al., 2018b; Pérez-Palacios et al., 2017; Fantazzini et al., 2009; García-García et al., 2019; Hansen et al., 2008; Pérez-Palacios et al., 2010; Pérez-Palacios et al., 2011; Pérez-Palacios et al., 2014; Lee et al., 2015; Monziols et al., 2006; Hendrick, 2005; Stark & Bradley, 1999; Antequera et al., 2007; Caballero et al., 2016).

Regarding feature extraction, higher correlation coefficients were achieved with the OPFTA in comparison with the GLCM for all the physicochemical parameters (Fig. 3). This result may reveal a better correlation between the physicochemical characteristics of the loins and the recurring patterns identified by the OPFTA than the correlation between the gray levels of the image counted by the GLCM. The structure of the muscles could be related to this hypothesis (Caballero et al., 2017b).

Apart from the correlation coefficient of the prediction results, the OPFTA is also more efficient from the point of view of computational complexity, mainly because it computes a lower number of features. Again, the OPFTA is slightly faster than the GLCM (22 vs. 46 ms, computed using a laptop INTEL i7-4510U, 2.6 GHz, and 16 GB RAM) (Caballero et al., 2018b).

According to this first experiment, where the OPFTA was selected as the best algorithm of feature extraction and SE-T1-weighted contrast was selected as the best MRI contrast for image acquisition, the second experiment was designed considering only OPFTA and SE-T1-weighted imaging. Two variables of LF-MRI sequence acquisition, TE and TR, were optimized in this second experiment. The objective was to maximize the correlation coefficient for the prediction of physicochemical parameters of fresh and dry-cured loins. The two full factorial CCDs involved 13 experiments, including 5 replicates of center points to verify any change in the estimation procedure and to measure the precision property. Table 3 presents the results of the 13 experiments in fresh loins, showing the complete CCD performed for the fresh loins of LF-MRI and the OPFTA as a feature extraction algorithm by using Design Expert v. 7 (Stat-Ease Inc., Minneapolis, Minnesota, U.S.A.).

Table 4 shows the results of the analysis of the variance by means of Fisher’s *F* test for some features of fresh and dry-cured loins. In the case of fresh loins, the *F*-values indicate the significance of the model for moisture and instrumental color L ^∗^, with a low chance (*p*-value), 0.003 and 0.007, respectively. Values lower than 0.05 were found for TR, TE × TR and TE^2^ for both parameters, indicating that they are significant terms. The lack of fit *F-values* of 7.48 and 64.34 (for moisture and instrumental color L ^∗^) are satisfactory and show that the model fits (remarks = S, significant). There is a low chance, *p*-value for a lack of fit of 0.041 and 0.001, for moisture and instrumental color L ^∗^, respectively, that a very large lack of fit *F*-value could occur due to noise. Moreover, additional parameters were checked for moisture and instrumental color L ^∗^ to verify this previous assumption. To corroborate the results, the predicted R^2^ coefficients for moisture and instrumental color L ^∗^were also computed, achieving values of 0.820 and 0.8950, whereas the adjusted R^2^ coefficients were 0.758 and 0.787, respectively. These results were consistent with the previous results. Additionally, the ratios for moisture and instrumental color L ^∗^ (12.159 and 10.280) indicated an adequate signal. Therefore, the response surface quadratic models for moisture and instrumental color L ^∗^ were adequate and significant.

**Table 4:** Analysis of variance for response surface model for the correlation coefficient (r) of the predicted physico-chemical characteristics of fresh and dry-cured loins (**S**: Significant/**NS**: Not significant).

			Model	TE	TR	TE x TR	TE^2^	TR^2^	Lack of fit
Fresh pork loins	aW	*F-value*	2.29	4.48	0.65	2.42	3.55	0.02	0.07
		*p-value*	0.155	0.022	0.447	0.164	0.102	0.884	0.606
		Remarks	**NS**						**S**
	pH	*F-value*	3.40	0.05	0.63	10.63	5.68	0.75	1.77
		*p-value*	0.071	0.828	0.454	0.014	0.049	0.414	0.292
		Remarks	**NS**						**NS**
	Moisture Content (%)	*F-value*	11.93	3.95	7.30	19.75	25.20	0.04	7.48
		*p-value*	**0.003**	0.087	0.035	0.003	0.001	0.853	0.041
		Remarks	**S**						**S**
	Lipids Content (%)	*F-value*	3.37	5.33	0.98	4.16	5.77	0.04	0.62
		*p-value*	0.072	0.054	0.356	0.081	0.047	0.842	0.637
		Remarks	**NS**						**NS**
	Color (L ^∗^)	*F-value*	8.54	1.80	5.67	17.90	15.58	0.61	64.34
		*p-value*	**0.007**	0.222	0.049	0.004	0.006	0.786	<0.001
		Remarks	**S**						**S**
Dry-cured loins	aW	*F-value*	0.82	2.32	0.14	0.93	0.72	0.06	0.93
		*p-value*	0.571	0.171	0.720	0.368	0.425	0.809	0.504
		Remarks	**NS**						**NS**
	pH	*F-value*	2.85	4.81	0.74	2.68	0.16	5.73	1.29
		*p-value*	0.102	0.064	0.417	0.145	0.700	0.048	0.393
		Remarks	**NS**						**NS**
	Moisture Content (%)	*F-value*	1.76	2.82	0.23	1.63	0.04	3.24	2.56
		*p-value*	0.239	0.137	0.647	0.243	0.852	0.114	0.192
		Remarks	**NS**						**NS**
	Lipids Content (%)	*F-value*	0.60	1.69	0.16	0.01	0.71	0.08	1.71
		*p-value*	0.705	0.235	0.703	0.918	0.428	0.791	0.301
		Remarks	**NS**						**NS**
	Color (L ^∗^)	*F-value*	1.30	2.47	2.58	1.22	0.21	0.02	0.70
		*p-value*	0.363	0.160	0.152	0.305	0.661	0.897	0.600
		Remarks	**NS**						**NS**
	Salt Content (%)	*F-value*	1.43	1.05	4.19	0.57	0.77	1.06	3.25
		*p-value*	0.321	0.341	0.080	0.474	0.410	0.337	0.142
		Remarks	**NS**						**NS**

Regarding the dry-cured loins, the model F-values of 0.82, 2.85, 1.76, 0.60, 1.30 and 1.43 for water activity, pH, moisture, lipid content, instrumental color L ^∗^, and salt content, respectively, implied the insignificance of the models for these parameters. This lack of fit indicates the absence of a functional relationship between TE and TR and the response variables.

The differences between fresh loins and dry-cured loins and among physicochemical parameters regarding the significance of the model prediction can be related to the content of water in the samples. T1-weighted MRI techniques allow the detection of hydrogen, which lengthens the T1 relaxation time (Lufkin, 1998). Therefore, the decrease in the water content during meat product processing will modify hydrogen detection. Therefore, the higher percentage of moisture content in fresh hams and the relationship between this parameter and the luminosity (instrumental color L ^∗^) of meat samples may explain the results of this study.

**Figure 4:**
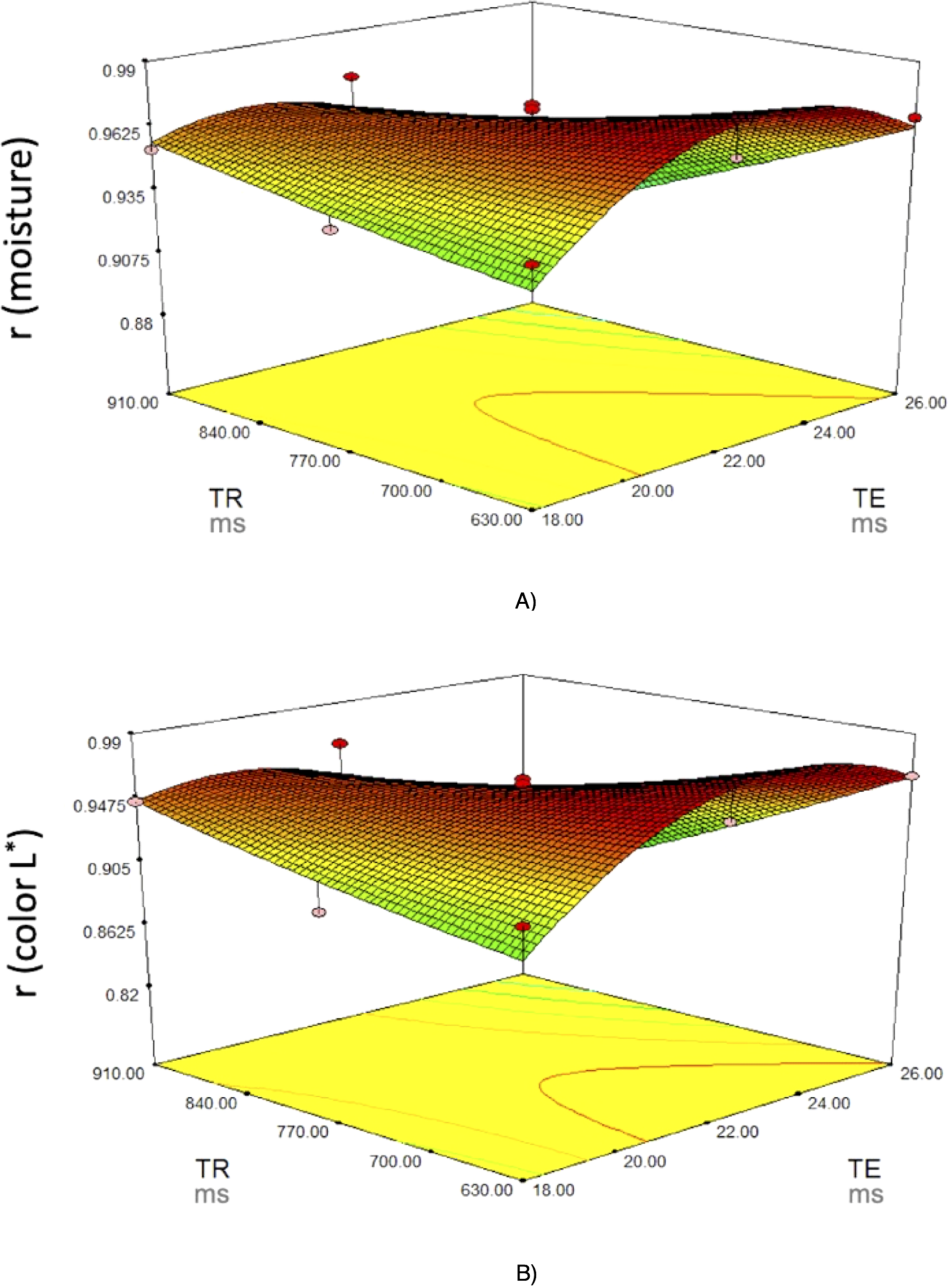
Response surface plots for (A) moisture and (B) color L^∗^. Response surface plots on the correlation coefficients (r for the predicted physico-chemical parameters) of fresh pork loins as affected by the MRI acquisition parameters (Echo Time (TE) and Repetition time (TR)).

Figure 4 shows the surface and contour plots for each significant response function (r of moisture and instrumental color L ^∗^) as affected by two variables (TE and TR) for fresh loins. It can be observed that the correlation coefficients of moisture and instrumental color L ^∗^ increased as the TR rose, and that the highest correlation was obtained with the lowest TR and medium-to-high TE.

To select the optimum values for these variables, the criteria applied were to be in the range for TE and TR and to maximize the correlation coefficients for all physicochemical parameters analyzed. The best solution indicated 22 and 630 ms for TE and TR, respectively, which may achieve very good to excellent correlation coefficients (*r* = 0.97−0.98) for all the characteristics.

### Quality characteristics based on the physico-chemical dataset

Table 5 shows the results of the physicochemical analyses of the experimental batch of fresh and dry-cured loins. The results are consistent with previous studies (Caballero et al., 2020; Kim & Kim, 2018; Menéndez et al., 2018; Muriel et al., 2004; Pérez-Palacios et al., 2019; Utrilla, Soriana & Ruiz, 2010). The differences in these parameters between fresh loins and dry-cured loins are attributed to the curing process, which leads to a loss of water. This water loss causes a decrease in moisture, water activity and color coordinate L ^∗^ from fresh to dry-cured loins (Muriel et al., 2004).

**Table 5:** Values of quality parameters (mean and standard deviation) of fresh and dry-cured loins determined by physico-chemical analyses and predicted by applying the optimized MRI parameters.

Feature	FRESH		DRY-CURED	
	Physico-Chemical	Predicted	Physico-Chemical	Predicted
Water activity (aW)	0.97 ± 0.01	0.97 ± 0.01	0.88 ± 0.01	0.88 ± 0.01
pH	5.54 ± 0.02	5.54 ± 0.01	5.85 ± 0.05	5.86 ± 0.04
Moisture content (%)	72.01 ± 2.76	72.05 ± 1.46	42.13 ± 2.46	42.97 ± 1.75
Lipids content (%)	6.04 ± 0.63	6.04 ± 0.47	6.10 ± 0.51	6.14 ± 0.45
Instrumental color L ^∗^	48.43 ± 0.44	48.46 ± 0.06	37.42 ± 1.54	37.44 ± 1.16
Salt content (%)	—	—	2.92 ± 0.13	2.91 ± 0.06

### Validation of the predictive model

When the procedure to predict the physicochemical characteristics of pork loins by using LF-MRI was optimized, it was also verified considering the validation batch of loins (Fig. 1). First, the evaluation metrics for the estimated features and validation batch parameters were evaluated for fresh loins (Table 5); high correlations were obtained for the estimated parameters and validation batch.

Tables 6 and 7 show several quality measures (including the correlation coefficient r for the prediction model and validation batch), which were calculated to validate the optimization procedure in fresh (Table 6) and dry-cured loins (Table 7).

**Table 6:** Quality measures (r, RMSEP, WAPE, TSTD and MAE) of the physico-chemical parameters of fresh loins, predicted by applying the optimized MRI parameters.

Quality measure	Water activity (aW)	pH	Moisture content (%)	Lipids content (%)	Instrumental color L*
r predicted	0.979	0.950	0.966	0.978	0.955
r from validation	0.978	0.959	0.971	0.984	0.953
RMSEP	0.001	0.001	0.018	0.065	0.001
WAPE	0.001	0.001	0.019	0.067	0.001
TSTD	0.002	0.009	1.434	0.477	0.064
MAE	0.001	0.008	1.300	0.393	0.059

**Table 7:** Quality measures (r, RMSEP, WAPE, TSTD and MAE) of the physico-chemical parameters of dry-cured loins, predicted by applying the optimized MRI parameters.

Quality measure	Water activity (aW)	pH	Moisture content (%)	Lipids content (%)	Instrumental color L*	Salt content (%)
r predicted	0.949	0.892	0.851	0.865	0.893	0.932
r from validation	0.958	0.891	0.853	0.881	0.878	0.934
RMSEP	0.003	0.007	0.005	0.073	0.004	0.022
WAPE	0.005	0.008	0.008	0.082	0.006	0.024
TSTD	0.006	0.042	1.496	0.521	0.186	0.085
MAE	0.002	0.040	1.348	0.447	0.157	0.063

The correlation values (r) were higher than 0.75 for all physicochemical parameters of fresh and dry-cured loins, which indicates a very good to excellent correlation (Colton, 1974). The WAPE and RMSEP were lower than 0.01% in all the cases. For all quality parameters, the TSTD values are slightly higher than the MAE values, indicating a lower dispersion in the computer prediction than in the true measurements.

The values obtained by physicochemical analysis and predicted by applying the optimized LF-MRI procedure are compared in Table 5. No differences were found for any of the physicochemical characteristics of fresh and dry-cured loins.

These results support the appropriateness of the LF-MRI optimization procedure to determine the physicochemical parameters of loins in a nondestructive way.

## Conclusions and Future Works

This research is the first study that has specifically optimized LF-MRI acquisition for the evaluation of the physicochemical characteristics of fresh and dry-cured loins.

The tunable parameters relaxation times (T1 and T2), TR, and TE of the image acquisition of fresh and dry-cured loins were optimized and validated in LF-MRI in this study, allowing us to determine the physicochemical characteristics of loins with high accuracy in a nondestructive way.

The influence of MRI contrast (SE-T1-weighted vs. SE-T2-weighted), TE and TR on the determination of the physicochemical parameters of loins has been suggested. This effect is more notable in fresh loins than in dry-cured loins. In addition, significant differences between most algorithms employed for MRI feature extraction (GLCM and OPFTA) in the accuracy of the analysis of the loins have been presented.

The use of SE-T1-weighted MRI contrast, TE and TR of 22 and 630 ms, respectively, for MRI acquisition, and OPFTA for MRI feature extraction achieved the most feasible and accurate results and appropriate values of the validation parameters. The optimization procedure could be applied to other LF-MRI systems or for other purposes by following the procedure proposed in this paper.

Our experiments were performed on meat products to analyze the quality parameters of loins in a nondestructive way. The authors of this paper are working with other meat samples, such as beef and chicken, and the optimization procedure will be repeated step by step with these new samples. In future works, other samples could be utilized, and a comparative study of the optimization process for different products of food technology (meat, fish, vegetables, fruit, etc.) could be presented.

##  Supplemental Information

10.7717/peerj-cs.583/supp-1 Supplemental Information 1 Raw data used in the experiments

## Additional Information and Declarations

### Data Availability

The following information was supplied regarding data availability: An example of the proposed algorithms and all the source code is available at GitHub: https://github.com/UniversidadExtremadura/Example-Volume-Extractor.
